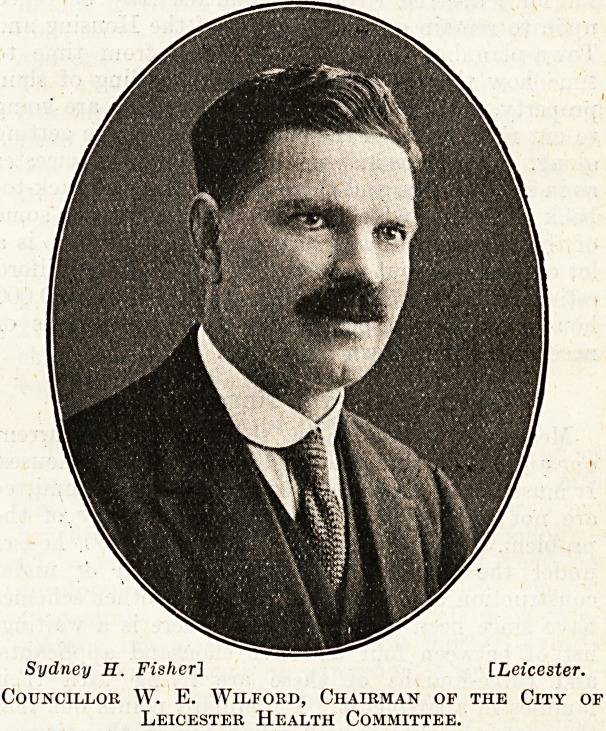# The Public Health: Interviews with Local Authorities—Leicester

**Published:** 1923-09

**Authors:** 


### THE PUBLIC HEALTH.

### INTERVIEWS WITH LOCAL AUTHORITIES.

### XII.—THE CITY OF LEICESTER.

THERE is only one possible note on which to begin a review of the health of the City of Leicester written at the present time. It is that of the surely unique record of long and faithful service in the cause of the public health registered by the retirement of Alderman Windley, in November last, from the chairmanship of the Health Committee, after forty-six years in office. The services of this distinguished public health veteran have been appropriately recognised by the foundation of four Secondary School scholarships, named after him, as the result of a testimonial fund raised by public subscription. In the forefront of a movement of recognition of what Dr. Killick Millard, the Medical Officer of Health, calls Alderman Windley's "long and wonderful term of office," was to be found his successor, Councillor W. E. Wilford. To him and to Dr. Millard, and to the Assistant Medical Officer of Health, Dr. Wyville Thomson (the Tuberculosis Officer for the city) we are indebted for a review of the health conditions of the city to which it is difficult to do justice within the limits of a single article.

Alderman Windley, late Chairman of Leicester Public Health Committee.

*Sydney H. Fisher*] [*Leicester.*

Councillor W. E. Wilford, Chairman of the City of Leicester Health Committee.

### A Proud Record.

In this series of articles we have been, and shall continue to be, sparing of statistics. But it is in the tables of figures which the Medical Officer so faithfully compiles that are happily to be found the milestones of progress. Alderman Windley, looking back over half a century, and then coming down to the day on which he vacated the chair, finds a population doubled, a death-rate halved, an infantile mortality rate divided by three. It is a proud record. But it would be a bad thing for Leicester if the new chairman and the medical officer were inclined to be complacent about it all. This they most emphatically are not. They can still find an abundance of work ready to hand. The chairman of the Health Committee is first and foremost a fresh-air man. He recruits on the golf links the energies which find such free play in committee. But he wants everyone in Leicester to play golf, or cricket, or tennis, or find some recreation in the open air. Leicester is exceptionally fortunate in her parks and open spaces. The wonderful stretch of lawns and other well-kept grass which comes down from the thirteenth century as a heritage from old Simon de Montfort—Sir Simon the King the common people called him—and a vast multitude listening on a lovely summer evening to the band of the Coldstream Guards and forgetting all about the noise and vibration of the hosiery workshops, or the three hundred and fifty processes which go to the manufacture of a pair of boots—these have left on our minds a very pleasant impression of some of the amenities which the people of Leicester enjoy.

### Tennis *v.* Tuberculosis.

The Council see to it that there are well-groomed lawns, beautiful flower-beds, municipal golf-links, cricket-pitches, bowling-greens, tennis-courts. Very good, says Mr. Wilford to the Council, but give us more of it, lots more: we want hundreds of tennis-courts; and he proceeds to demonstrate that each tennis-court costs less than the keep of a consumptive patient for a year. No Health Committee is worth its salt unless it is inspired with a divine discontent. And we find in the will-to-progress of Councillor

Wilford and his committee, who are fortunate in their medical advisers, a guaranty for the good government of Leicester so far as health is concerned. A drop, in a half-century, in the infant mortality, they say, from 242 (a terrible figure) to 87 is a very satisfactory record; but 87 won't do. It is itself an average for different wards of the city, and in one slum ward it is as high as 140. Therefore they are determined that the vigorous campaign for getting old and slum property overhauled and put into proper repair, which was embarked upon before the war, shall be resumed and conditions of life made more tolerable. And, fresh from visits which they had made to some of the overcrowded areas—Councillor Wilford is a great believer in seeing things for himself,—the Health Committee may be relied upon to remain on the doorstep of the Housing and Town-planning Committee to know from time to time how their schemes for the rebuilding of slum property, and for the arterial roads which are going to cut right through the slums in places, are getting along. The slums may not be so obvious in Leicester as in some places, and there are not the bad back-to-back conditions which make so sorry a story in some of the industrial towns further north, but there is a lot of old, worn-out property, and Councillor Wilford estimates that not less than 20,000 out of 50,000 houses are crying out for long-overdue repairs or needing to come down altogether.

### Marriage and the Housing Famine.

Meantime, as elsewhere, there is the concurrent question of an actual serious scarcity of new houses. It must not be supposed that the Housing Committee are not fully alive to the extreme urgency of the problem. There were, in fact, some 800 houses under the Council schemes constructed or under construction at the end of 1922, and other schemes have since been approved. But there is a waiting-list of between four and five thousand applicants, and three-fourths of these are living with their families in apartments. Dr. Millard points out that the marriage rate is still much higher than it was before the war, and that, so far from young people being deterred from matrimony by the housing scarcity, this is actually having the opposite effect. In normal times it was the custom for young people to defer marriage until they could start a home of their own. This involved saving up for furniture, etc. To-day, owing to the virtual impossibility of obtaining a house, it is the custom to get married without waiting to save up. That the removal of this obstacle to marriage is an advantage is, however, very doubtful.

### The Care of the Consumptive.

We do not propose to inquire whether the unduly high incidence of phthisis amongst boot and shoe operatives can be attributed to factory or to home conditions. The Chairman of the Health Committee considers that it is to the latter rather than the former we must look; certain it is that many of the factories are well-lit, well-ventilated modern buildings. The subject was specially investigated in 1915 by the Medical Research Committee, who, among other recommendations, suggested the establishment of a sanatorium to which a boot and shoe factory should be attached, for all consumptives in the industry who would otherwise be left by the wayside. It is a problem full of difficulties, social and economic, to which Mr. Wilford and his committee might well devote their energies. Mr. Wilford himself is quite definite in his confirmation of views which we have seen expressed elsewhere, that any attempt to provide under ordinary economic conditions in a boot factory for a man suffering from tuberculosis is foredoomed to failure. The division and sub-division of labour is too great, and each man is an indispensable cog in the wheel.

### A Welfare Superintendent.

They have, at any rate, taken a new and experimental step in Leicester in the appointment of a Welfare Superintendent at the Groby Road Sanatorium. His duties are to organise gardening and other outdoor occupations, physical exercises, field rambles and entertainments, and generally to assist the patients in living cheerful and interested lives and to get outside what has too often been the dull routine of sanatorium life. We may express the hope that this interesting experiment will be followed by other authorities, and that Leicester will lead the way, or do so jointly with the other principal boot and shoe areas, in organizing remunerative occupation for consumptive workpeople in their own trades and not too far away from their own homes.

### The Need for After-care.

Leicester is having a health-week shortly, and at the time of our visit Councillor Wilford was busy arranging for a special day to be set apart for propaganda and a collection to raise money to be specially applied to the after-care of consumptive patients. This is of the utmost importance, and, indeed, without adequate attention to this matter of after-care, a great deal of the cost and care of sanatorium treatment is wasted. After-care lends itself peculiarly well to organised voluntary effort, and it is to be hoped that the endeavours which the committee will make to enlist public interest and support will meet with a large and enthusiastic success. Allied to the general question of after-care is the special need for an open-air school, and the Health Authorities are pressing this on the Education Committee. It would be especially valuable to children who had completed a course of treatment at the children's sanatorium, Anstey Lane, who are not strong enough on returning home to work at school under normal conditions.

### An Unvaccinated City.

Leicester is practically an unvaccinated city. The returns show that 173 children only were vaccinated in 1922, while 4,286 exemptions were granted, and that during the past thirty-eight years not more than 10 per cent. of the children have been vaccinated. We record this, and the fact that during the last eighteen years there has only been one single case of small-pox in Leicester (an imported case in 1913), without comment. It should be stated that Dr. Millard is not only ready to tackle small-pox, if unhappily the need should arise, in his own city, but had, just prior to our visit, sacrificed his holiday to go, with the goodwill of his committee, to the assistance of Gloucester

### Various Activities.

Space forbids more than a mere reference to many other features of the health conditions in this old city of Leicester which looks so modern and so clean, but which, as we have seen, harbours much old property. Excellent work is being done by the Leicester Health Society at the twelve schools for mothers (" Babies' Welcomes " they call them), the Corporation having wisely decided to co-operate with and support, rather than supplant, the voluntary body in the working of the Maternity and Child Welfare scheme. The Maternity Home in Westcotes Drive has been kept busy, and the vacant St. Martin's Vicarage, with its airy and commodious rooms and playground for the toddlers, has proved an excellent investment since it was rented for a day nursery in 1922. Mention must also be made of the work accomplished by the Poor Boys' and Girls' Summer Camp at Maplethorpe, where the splendid figure of 13,323 has been reached as the number of children who have benefited since the institution was started twenty-five years ago.

### Sleeping Dogs.

The increasing recognition of the T.B. dispensary as a centre of diagnosis, the issue of a cancer leaflet, the movement in favour of clean milk (the committee are having Grade A milk supplied to the institutions under their control), are further evidences of the " liveliness " of the Health Committee and the Medical Officer and his staff. A recent excursion on the part of Councillor Wilford to the haunts of some of the street ice-cream vendors, where some pretty bad conditions were discovered, is evidence, too, that comparatively minor matters are not overlooked. Leicester, as we have said, is a fine clean city. There are sleeping dogs there, as elsewhere, which, so far as they are a menace to the health of the community, Councillor Wilford and Dr. Millard are by no means disposed to " let lie " merely because they will give them trouble. Trouble is all in a day's march with them, and they take it in their stride.

### ENDEMIC MALARIA IN ENGLAND.

ONE of the many sinister by-products of the war is a very considerable increase in the number of cases of malaria in England, France, Germany and other countries where malaria has hitherto been almost exclusively limited to travellers returning from the Tropics. Now it would seem that the thousands of malaria-infected soldiers who have returned to their homes in Europe have transmitted this disease to home-bred mosquitoes, and their capacity for passing malaria on to a healthy person is the greater because they are not generally suspect. This problem has recently been under discussion in the German medical press, and it is a noteworthy fact that among a series of 38 cases of endemic malaria, only two of the patients were women. This comparative immunity of women to endemic malaria is probably associated with their indoor life. This matter is not one for scare-mongering, but it would be well if a census of cases of endemic malaria in England were to be taken and kept up to date.

## Figures and Tables

**Figure f1:**
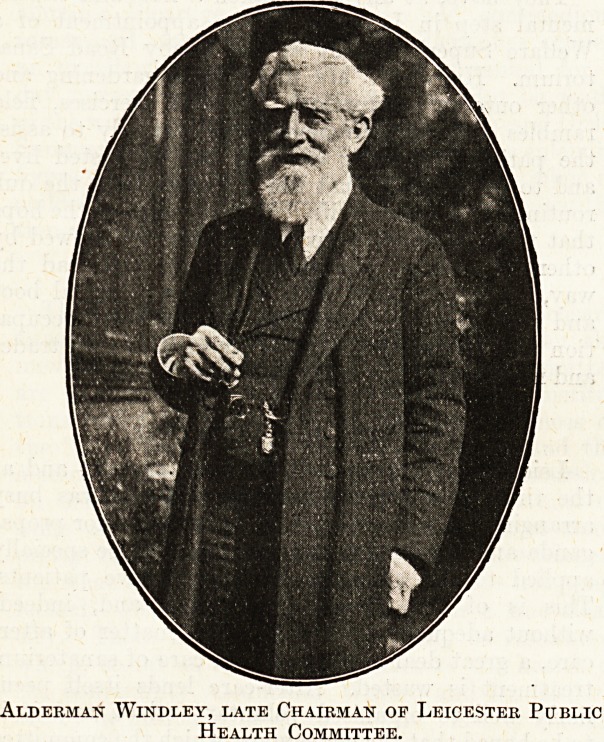


**Figure f2:**